# Supplementary material for: Electrografting of 4-Carboxybenzenediazonium on Glassy Carbon Electrode: The Effect of Concentration on the Formation of Mono and Multilayers
Source: Molecules. 2020 Oct 7;25(19):4575. doi: 10.3390/molecules25194575 (PMC7582525; doi:10.3390/molecules25194575)
Supplement: Supplementary file 1 [file molecules-25-04575-s001.pdf]

molecules

MDPI

Supplementary Materials: Electrografting of 4-carboxybenzenediazonium: the effect of concentration on the formation of mono and multilayers

Sereilakhena Phal[a], Kenichi Shimizu[a], Daniel Mwanza[b], Philani Mashazi[b,c], Andrey Shchukarev[a], Solomon Tesfalidet[a,*]

[a] Department of Chemistry, Umeå University, SE 90187 Umeå, Sweden

[b] Department of Chemistry, Rhodes University, Grahamstown, 6140 South Africa

[c] Institute for Nanotechnology Innovation Centre, Rhodes University, Grahamstown, 6140 South Africa

*Corresponding author: E-mail address: solomon.tesfalidet@chem.umu.se

XPS of electrografted GCE surface

The XPS measurements were performed on bare GCE and grafted GCE (CP/GCE). The grafting was made using CV at two concentrations of 4-CBD: 0.15 mmol.L^{-1} (1 and 22 cycles) and 2.50 mmol.L^{-1} (1 and 3 cycles). XPS survey spectra, Fig. S1, displays two main C 1s and O 1s photoelectron peaks and additional N 1s line of low intensity. The corresponding atomic concentrations are presented in Table S1. Comparison of survey spectra obtained for bare GCE and CP/GCE grafted with 2.50 mmol.L^{-1} 4-CBD (1 cycle) reveals that for the CP/GCE, the peak of C 1s decreased while the peak for O 1s increased compared to bare GCE. This leads to higher ratio of O 1s/C 1s for CP/GCE (0.182) compared to GCE (0.077). However, when GCE was grafted using 0.15 mmol.L^{-1} 4-CBD (1 cycle), the spectra did not show significant difference in the atomic concentrations of C 1s and O 1s, for which the ratio of O 1s/C 1s was 0.086 compared to 0.077 for the bare. When the grafting was performed using 0.15 mmol.L^{-1} 4-CBD (22 cycles) the ratio O1s/C1s increased to 0.199 which is close to the ratio obtained for grafting with 2.50 mmol.L^{-1} 4-CBD, 1 cycle (0.182). For grafting with 3 cycles using 2.50 mmol.L^{-1} 4-CBD the ratio of O 1s/C 1s was 0.206, which is not different from the values obtained for 1 cycle. These results indicate that grafting for 1 cycle using 0.15 mmol.L^{-1} is not enough to form a detectable amount of CP moieties at the surface. A multilayer is probably formed when the grafting is made using higher concentration of 4-CBD (1 cycle) or multiple scans (22 cycles) using the lower concentration.

The high-resolution spectra of C 1s and O 1s for bare GCE, CP/GCE grafted using 0.15 mmol.L^{-1} 4-CBD (22 cycles), and 2.50 mmol.L^{-1} 4-CBD (1 cycle) are presented in Fig. S2. The C 1s high-resolution spectrum was fitted with four components at 284.2 eV (sp^2 C in GCE), 285.4 eV (C-OH, C-O-C), 287.0 eV (C=O) and 288.6 (O-C=O). In Fig. S2, the two components at 284.2 and 288.6 eV are indicated for comparison. The main component at 284.2 eV (sp^2 C) decreased from 100% on bare GCE to 93% on CP/GCE for the lower concentration, 0.15 mmol.L^{-1} (22 cycles) and to 94% for the higher concentration, 2.50 mmol.L^{-1} (1 cycle). The decrease in the peak intensity for CP/GCE may be due to the formation of a thick layer that shields the carbon sp^2 from the GCE. For CP/GCE, another component that appeared at 288.6 eV corresponds to carboxylic group, HO-C=O [1]. This component accounted for 7 atomic % when the grafting was made with 0.15 mmol.L^{-1} (22 cycles) and 6 atomic %

for 2.50 mmol.L^{-1} (1 cycle). This peak, which was absent for the bare GCE, confirms that the GCE surface was successfully grafted with carboxyphenyl to almost the same extent.

The high-resolution spectra of O 1s for the grafted GCE also displayed two components, corresponding to the carboxylic functional group in the grafted layers. For CP/GCE grafted with 0.15 mmol.L^{-1} (22 cycles) the components appeared at 533.1 eV for O=C-O-H (50%) and 531.6 eV for O=C-O-H (48%) [1, 2]. The corresponding components for CP/GCE grafted with 2.50 mmol.L^{-1} (1 cycle) appeared at 533.0 eV for O=C-O-H (56%) and 531.5 eV for O=C-O-H (39%) [1, 2]. The intensity ratio of these two components is close to 1.1. This is another confirmation that the carboxyphenyl layer obtained from 4-CBD is grafted on the GCE.

Table S1. Atomic compositions on bare GCE and grafted GCE (CP/GCE) using 0.15 mmol.L^{-1} and 2.50 mmol.L^{-1} 4-CBD at different grafting cycles.

**Electrode**	**Atomic concentration / (at.%)**		
	**C1s**	**O1s**	**N1s**
Bare GCE	91.4	7.0	1.6
CP/GCE, 0.15 mmol.L^{-1} 1 cycle	92.1	7.9	-
CP/GCE, 0.15 mmol.L^{-1} 22 cycles	83.4	16.6	-
CP/GCE, 2.5 mmol.L^{-1} 1 cycle	83.5	15.2	1.4
CP/GCE, 2.5 mmol.L^{-1} 3 cycles	81.5	16.8	1.7

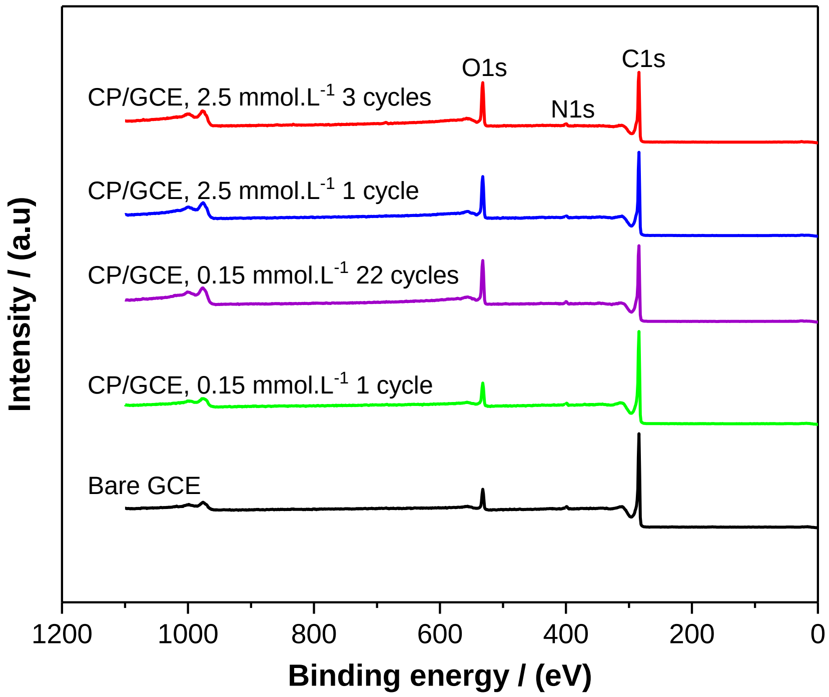

Fig. S1. Survey spectra of bare GCE (black), grafted GCE (CP/GCE) using 0.15 mmol.L^{-1} 4-CBD: for 1 cycle (green) and 22 cycles (violet), and grafted GC (CP/GCE) using 2.50 mmol.L^{-1} 4-CBD: for 1 cycle (blue) and 3 cycles (red).

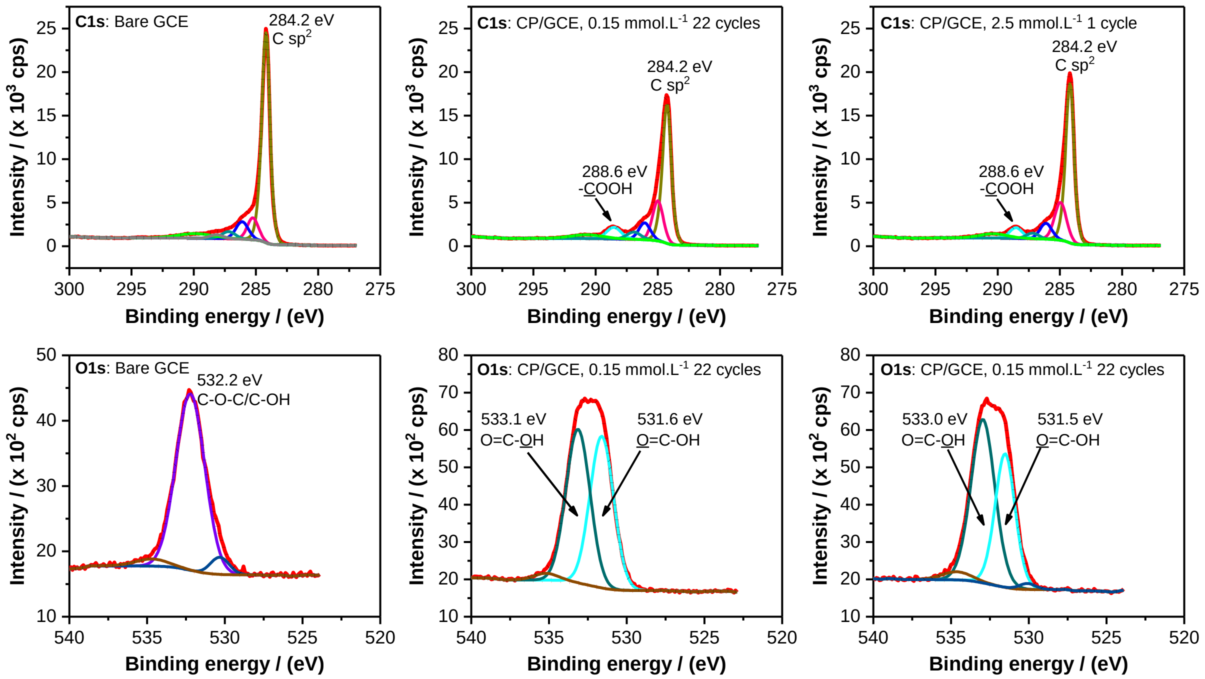

Fig. S2. High-resolution spectra of C 1s and O 1s for bare GCE and CP/GCE grafted using 4-CBD: 0.15 mmol.L^{-1} (22 cycles) and 2.50 mmol.L^{-1} (1 cycle).

References

1. Kam, C. S.; Leung, T. L.; Liu, F.; Djurišić, A. B.; Xie, M. H.; Chan, W.-K.; Zhou, Y.; Shih, K. Lead removal from water – dependence on the form of carbon and surface functionalization. *RSC Advances* **2018**, 8, 18355-18362.
2. Fan, L.-Z.; Qiao, S.; Song, W.; Wu, M.; He, X.; Qu, X. Effects of the functional groups on the electrochemical properties of ordered porous carbon for supercapacitors. *Electrochim. Acta* **2013**, 105, 299-304.
